# Scolytinae in hazelnut orchards of Turkey: clarification of species and identification key (Coleoptera, Curculionidae)

**DOI:** 10.3897/zookeys.710.15047

**Published:** 2017-10-19

**Authors:** Celal Tuncer, Milos Knizek, Juri Hulcr

**Affiliations:** 1 Ondokuz Mayis University, Faculty of Agriculture – Department of Plant Protection, Samsun, Turkey; 2 Forestry and Game Management Research Institute, Jiloviste – Strnady, Praha 5 – Zbraslav, CZ – 156 00, Czech Republic; 3 School of Forest Resources and Conservation, University of Florida, Gainesville, FL 32611, USA

**Keywords:** ambrosia beetles, bark beetles, pests

## Abstract

Hazelnut, a very important cash crop in Turkey, is frequently colonized by bark and ambrosia beetle species (*Scolytinae*). Some scolytine species may cause economic damage while other species do not; therefore, proper identification is important in orchard management. Extensive sampling demonstrated that the most common pest species in Turkey’s hazelnut orchards are *Anisandrus dispar*, *Xylosandrus germanus*, and *Xyleborinus saxesenii. Hypothenemus eruditus* can also be common, but only colonizes branches that are already dead. *Lymantor coryli*, *Hypoborus ficus*, *Taphrorychus ramicola*, and *Taphrorychus hirtellus* are rare and do not causes damage to live plants. *Xyleborinus saxesenii* appears to have been frequently misidentified and misreported as either *L. coryli* or *Xyleborus xylographus*. The former is rare, and the latter probably does not occur in Turkey. To avoid future misidentifications, a dichotomous identification key is provided for bark and ambrosia beetles of hazelnut orchards in Turkey.

## Introduction

Turkey is the world’s largest hazelnut producer, supplying nearly 80% of the total global production. The plantations in Turkey occupy nearly 690,000 ha (Anonymous 2013), produce 430,000 – 800,000 tons/year (TUIK 2011), and generate approximately 2 billion USD (Anonymous 2016). It is one of the primary cash crops for many farmers, especially in the Black Sea region. Hazelnut crops are also beneficial in that they protect the land against erosion.

Although Turkey is the world’s primary hazelnut producer, its productivity per area is lower than that of hazelnut-growing Western countries. Besides agronomic reasons, insect and mite pests appear to be a major impediment to efficient production. Hundreds of insect and mite species have been found to be pests in Turkish hazelnut orchards (Tuncer and Ecevit 1997), ten of which have been classified as significant pests in hazelnut production, including bark and ambrosia beetles.

Bark and ambrosia beetles (Coleoptera: Curculionidae: Scolytinae) are a well-known and diverse group of insects often capable of causing serious damage estimated in millions of US dollars (Knížek and Beaver 2007). They are also one of the major pest groups in Turkish hazelnut orchards (Tuncer and Ecevit 1997). Weakened hazelnut trees are frequently heavily infested and eventually killed by these insects, especially in orchards along the Black Sea coast where drainage problems occur (Tuncer and Ecevit 1997). Other factors that exacerbate bark and ambrosia beetle attacks include placement of hazelnut orchards close to forested areas, and placement on steep slopes; neither situation is typically managed well. Until today, six bark and ambrosia beetle species were reported from Turkish hazelnut orchards: *Anisandrus dispar* (Fabricius, 1792), *Hypothenemus eruditus* (Westwood, 1834), *Lymantor coryli* (Perris, 1855), *Xylosandrus germanus* (Blandford, 1894), *Xyleborinus saxesenii* (Ratzeburg, 1937) and *Xyleborus xylographus* (Say, 1826) (Işık et al. 1987; Ak et al. 2005a, b). Some of these species have also been found in hazelnut orchards in Italy, USA, and elsewhere (Anonymous 1935; Speranza et al. 2009). Because simple chemical control is not feasible due to the phenology and the cryptic nature of these insects (Ak et al. 2005a), it is important to develop a more sophisticated, integrated approach to the prevention of damage. A key step in the development of any integrated pest management program is accurate identification of the involved organisms.

Bark and ambrosia beetles are a diverse group of small insects with uniform morphology making them notoriously difficult to identify (Wood 1982). It appears that earlier reports on the identities of bark and ambrosia beetles in Turkish hazelnut orchards may have been erroneous. Early studies in Turkish hazelnut orchards claimed that there were four bark and ambrosia beetle species, *A. dispar*, *H. eruditus*, *L. coryli*, and *X. xylographus*, but lacked sufficient evidence to support such claims (Işık et al. 1987). Ak et al. (2005a, b) reported the same four species. Neither of these studies reported *X. saxesenii*, yet photographs of alleged *L. coryli* damage actually resemble damage inflicted by *X. saxesenii*. Ak et al. (2010) also recorded *L. coryli* as a new fruit pest of Kiwi using a photograph to support the claim. However, the photograph was actually of *X. saxesenii* and not *L. coryli*. *Xyleborinus saxesenii* was not identified correctly in studies carried out in Turkish hazelnut orchards until 2013 (Saruhan and Akyol 2013). Additionally, the identification of *X. xylographus* warrants scepticism because this species is distributed in the Nearctic region (Wood and Bright 1992; Knížek 2011); it is not possible to confirm studies (Işık et al. 1987; Ak et al. 2005) reporting occurrence of *X. xylographus* in Turkey, but it is highly probable that the specimens were misidentified *X. saxesenii* (Wood 1982) and the species does not occur in Turkey at all. Though Wood and Bright (1992) claim that *X. saxesenii* is a native species in Turkey, the lack of evidence concerning the species’ presence in previous studies involving hazelnut orchards strengthens the assertion that it was a misidentification. Ak et al. (2011) found two species, *A. dispar* and *X. germanus*, in kiwi orchards by ethanol trapping, establishing likely the first record of *X. germanus* in Turkey (Knížek 2011). Recently, more extensive study carried out by Tuncer et al. (unpublished 2012-2016) on bark and ambrosia beetles in hazelnut orchards revealed that *A. dispar, H. eruditus, Hypoborus ficus* Erichson,1836, *L. coryli, Taphrorychus hirtellus* Eichhoff,1878, *X. germanus* and *X. saxesenii* are present.

Without experience in identification, *X. saxesenii* tended to be mistaken for *L. coryli*, and *A. dispar* (male) for *X. germanus*. Frequent misidentification occurred whether the specimen was viewed under a microscope or with the naked eye and are especially troublesome during field studies. Therefore, to prevent future misidentifications of these species and to increase the efficiency of hazelnut pest management, a simple and easy identification key for bark and ambrosia beetles in hazelnut orchards is needed.

## Materials and methods

Examined material consisted of samples belonging to five species which were obtained from hazelnut orchards in the mid-Black Sea region. Specimens were collected with ethanol-baited traps as well as extracted from infested hazelnut trunks. Two species (*H. ficus* and *T. hirtellus*) were only obtained by excision directly from hazelnut wood. Though *T. ramicola* and *X. xylographus* were not sampled in this work, they were included in the key due to their presence in early records. *X. xylographus* was provided by the Museum of Entomology (FSCA) at the DDivision of Plant Industry (DPI) of the Florida Department of Agriculture and Consumer Services, Gainesville, FL, USA. Pictures used in this paper were taken using an Olympus SZX 16 stereomicroscope and Olympus DP72 camera, with STREAM BASIC 1.9 software. HELICON FOCUS 6.2.2 and HELICON FILTER 5.4 were used to stack photos for better depth of field. Studies were carried out in the Forest Entomology laboratory at the School of Forest Resources and Conservation, University of Florida (Gainesville, FL, USA), Department of Plant Protection in Ondokuz Mayis University (Samsun, Turkey), and Department of Forest Protection Service in Forestry and Game Management Research Institute (Jíloviště, Czechia). The nomenclature used by Wood and Bright (1992) as well as later taxonomic and systematic adjustments (Hulcr et al. 2007, Knížek 2011) are followed in this work. The measurement parameters of the species were taken from Pfeffer (1995) and Wood (1982).

## Results

A list of the bark and ambrosia beetles present in hazelnut orchards of Turkey is provided in Table 1 (in alphabetical order).

**Table 1.** Scolytinae species in hazelnut orchards of Turkey and their distribution in Turkey and in the World.

Species	Distribution in Turkey	World distribution
*Anisandrus dispar* (Fabricius, 1792)	Adana, Ankara, Artvin, Bartın, Bolu, Bursa, Çorum, Denizli, Duzce, Giresun, Gümüşhane, Hatay, İstanbul, Karabük, Kastamonu, Muğla, Niğde, Ordu, Rize, Sakarya, Samsun, Trabzon, Zonguldak, Western Mediterranien	Asia, Europe, Nearctic, Oriental
*Hypoborus ficus* Erichson, 1836	Adana, İstanbul, İzmir, Mersin, Sakarya	Asia, Europe, North Africa
*Hypothenemus eruditus* (Westwood, 1834)	Aydın, Mersin, Samsun	Afrotropical, Asia, Australia, Europe, Nearctic, Neotropical, North Africa, Oriental
*Lymantor coryli* (Perris, 1855)	Düzce, Samsun	Asia, Europe
*Taphrorychus hirtellus* Eichhoff, 1878	Hatay, İstanbul, Sakarya, Sinop	Asia, Europe, North Africa
*Taphrorychus ramicola* Reitter, 1895	Bartın, Hatay, Sakarya, Trabzon, Western Mediterranien,	Asia, Europe
*Xyleborinus saxesenii* (Ratzeburg, 1937)	Amasya, Antalya, Artvin, Bolu, Düzce, Giresun, Hatay, Isparta, İstanbul, Kocaeli, Konya, Mersin, Muğla, Ordu, Rize, Sakarya, Samsun, Sinop, Trabzon, Zonguldak	Afrotropical, Asia, Australia, Europe, Nearctic, Neotropical, North Africa, Oriental
*Xylosandrus germanus* (Blandford, 1894)	Duzce, Ordu, Samsun	Asia, Europe, Nearctic, Oriental

### Key to bark and ambrosia beetles from hazelnut orchards of Turkey

**Table:** 

1	Body shortly oval, stout, length-to-width ratio of pronotum 0.6, basal margin of elytra procurved, elevated and armed by marginal crenulations. 1.0–1.3 mm.	tribe ***Hypoborini*, *Hypoborus ficus*** (Figs 1–2)
–	Body elongated, cylindrical, length-to-width ratio of pronotum 0.9–1.1, basal margin of elytra straight, transverse, unarmed	tribes ***Cryphalini, Dryocoetini*** and ***Xyleborini***...**2**
2	Body covered with flattened setae or erect scales, particularly the elytral declivity; antennal club segments of approximately the same size, with a distinct partial septum (dark incision) (Fig. 5); 0.7–0.8 mm in ♂, 1.0–1.8 mm in ♀	tribe ***Cryphalini*, *Hypothenemus eruditus*** (Figs 3–5)
-	Body mostly shining, covered with fine setae which are not flattened, antennal club rounded, the first segment much more prominent than the second and third, septum absent, (Figs 6–7)	tribes ***Dryocoetini*** and ***Xyleborini***...**3**
3	First suture on the antennal club procurved, convex, the first segment round (Fig. 6) ; base of pronotum (area adjacent to elytra) coarsely and densely punctured	tribe ***Dryocoetini***...**4**
–	First suture on the antennal club recurved, concave, the first segment sickle-shaped (Fig. 7). Basal part of pronotum only finely and sparsely punctured, shining or reticulated	tribe ***Xyleborini***...**6**
4	Pronotum oval from dorsal view, convex with no distinct summit from lateral view, approximately first third asperate, posterior two thirds punctate, smooth and shining in between the punctures, hair-like setae missing in elytral and declivital disc, restricted only along the suture and lateral parts on elytral declivity. 1.6–2.2 mm	***Lymantor coryli*** (Figs 8–9)
–	Pronotum cylindrical from dorsal view and with an indicated summit from lateral view, first half asperate, posterior half punctate, smooth and shagreened, semi-shining in-between the punctures, elytral vestiture occurring on the whole surface of elytral disc and declivity	***Taphrorychus***...**5**
5	Pronotum convex with no distinct summit from lateral view; elytra shining, with clearly visible slightly impressed punctured striae. 1.2–2.0 mm	***Taphrorychus ramicola*** (Figs 10–11)
–	Pronotum clearly marked by summit in the middle from lateral view; elytra matt, without punctured impressed striae. 1.6–1.8 mm	***Taphrorychus hirtellus*** (Figs 12–13)
6	Robust; black or very dark brown when mature; the anterior margin of pronotum with a row of flat teeth (serrations)	**7**
–	Slender; orange to brown, if dark brown then elytra darker than pronotum; the anterior margin of pronotum without elevated teeth (only with asperities appressed to the surface);	**8**
7	Procoxae widely separated, the gap wider than antennal scapus (the first long segment) (Fig. 14); surface of the pronotal base shining (Fig. 15). 1.0–1.8 mm in ♂, 2.0–2.3 mm in ♀	***Xylosandrus germanus*** (Figs 14–17)
–	Procoxae only narrowly separated (Fig. 18); the gap not wider than scapus; surface of the pronotal base dull, reticulated, not shining (Fig. 19). 1.8–2.1 mm in ♂, 3.2–3.6 mm in ♀	***Anisandrus dispar*** (Figs 18–21)
8	Scutellum small, conical (“knob-like”), nearly concealed by a tuft of setae (Fig. 22); elytral declivity surrounded by small sharp denticles, but striae 1, 2, and 3 (spaces between rows of punctures) on the declivity without any denticles. 1.6–1.8 mm in ♂, 2.0–2.4 mm in ♀	***Xyleborinus saxesenii*** (Figs 22–25)
–	Scutellum triangular, flush with surface of elytra, easily visible; all striae on elytral declivity with uniform small dull granules in all striae, 1.9 mm in ♂, 2.3–2.7 mm in ♀	***Xyleborus xylographus*** (Figs 26–27)

**Figures 1–2.**
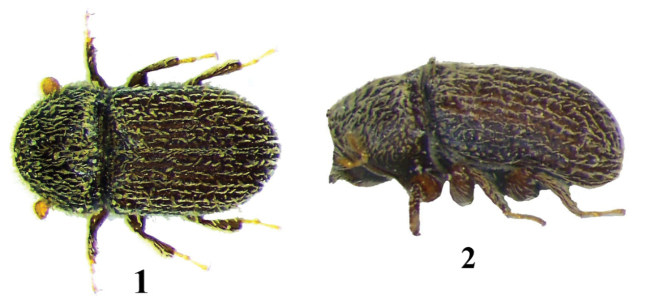
*Hypoborus ficus*, adult. **1** dorsal aspect **2** lateral aspect.

**Figures 3–4.**
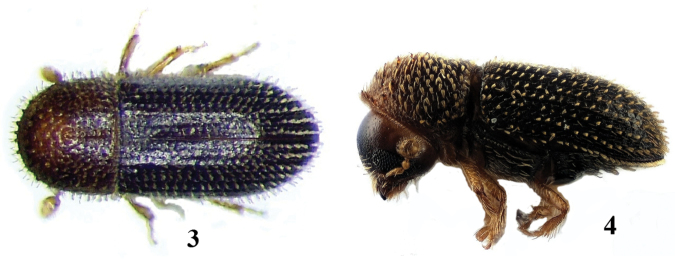
*Hypothenemus eruditus*, adult. **3** lateral aspect **4** dorsal aspect.

**Figures 5–7.**
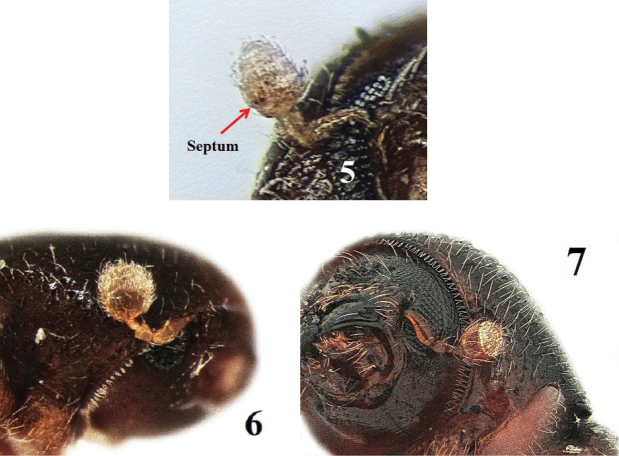
Antennal club. **5** *Hypothenemus eruditus* **6** *Lymantor coryli* **7** *Xylosandrus germanus*.

**Figures 8–9.**
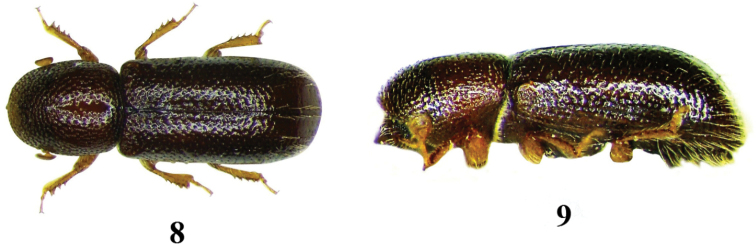
*Lymantor coryli*, adult. **8** dorsal aspect **9** lateral aspect.

**Figures 10–11.**
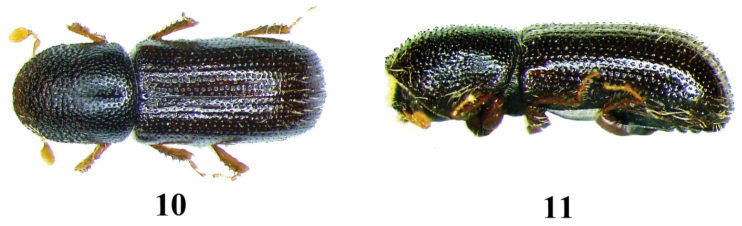
*Taphrorychus ramicola*, adult. **10** dorsal aspect **11** lateral aspect.

**Figures 12–13.**
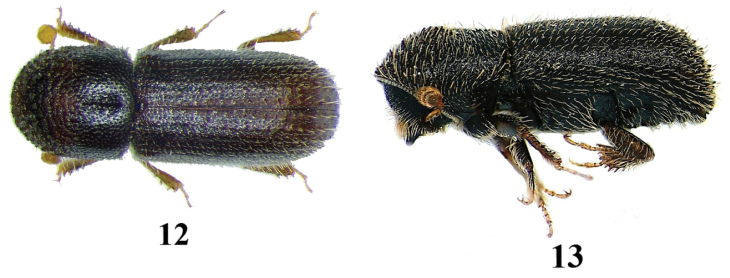
*Taphrorychus hirtellus*, adult. **12** dorsal aspect **13** lateral aspect.

**Figures 14–17.**
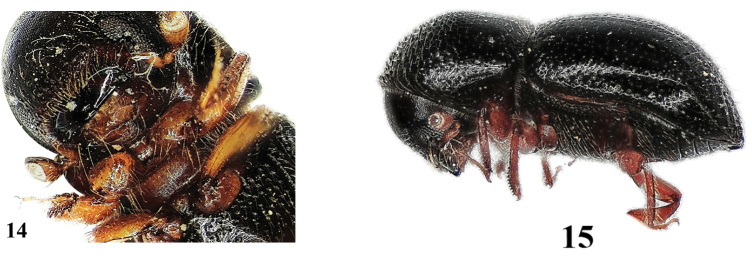
*Xylosandrus germanus*. **14** female separation of procoxa **15** female, lateral aspect **16** female, dorsal aspect **17** male, lateral aspect.

**Figures 18–21.**
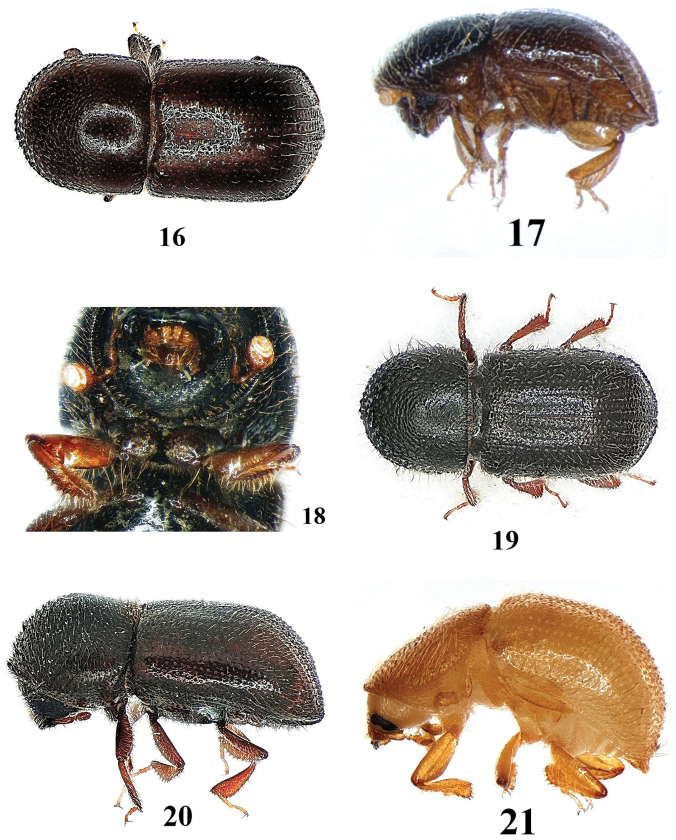
*Anisandrus dispar*. **18** female, separation of procoxae **19** female, dorsal aspect **20** female, lateral aspect **21** male, lateral aspect.

**Figures 22–25.**
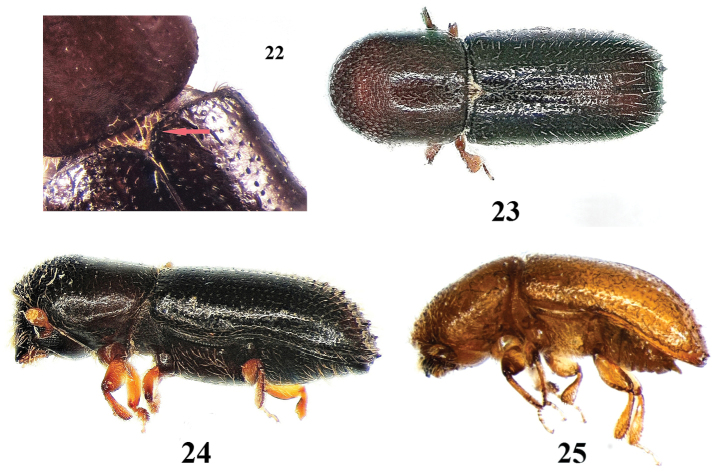
*Xyleborinus saxesenii*. **22** female, scutellum **23** female, dorsal aspect **24** female, lateral aspect **25** male, lateral aspect.

**Figures 26–27.**
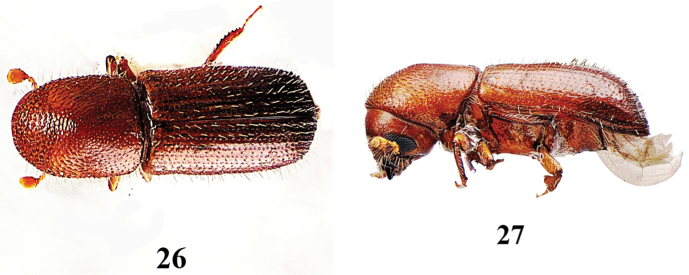
*Xyleborus xylographus*, female. **23** dorsal aspect **24** lateral aspect.

## Discussion

The sampling revealed that *A. dispar*, *X. saxesenii*, *X. germanus*, and *H. eruditus* are common in hazelnut plantations. In addition, a few specimens of *L. coryli, T. hirtellus*, and *H. ficus* were collected by examining the hazelnut tree trunks. It therefore appears that *A. dispar*, *H. eruditus*, *X. germanus*, and *X. saxesenii* are regularly found in hazelnut orchards, while *T. hirtellus* and *L. coryli* are not very common. *Hypoborus ficus* is a common species on fig trees in Turkey (Selmi 1998), and thus was probably an unusual occurrence in hazelnut. The reports of *X. xylographus* appear to be the result of repeated misidentification (Selmi 1998). We hope this identification key will help prevent future misidentifications of bark and ambrosia beetles in hazelnut and other orchards.

It is worth noting that several species treated here [*A. dispar*, *X. germanus* (Ak et al. 2011), and *X. saxesenii* (Ak et al. 2010)] were also recorded as pests in kiwi orchards, which are grown in the same region of Turkey as hazelnut. Hence, this identification key will also help with studies on kiwi insects.

Additional species are likely to be found on hazelnut in Turkey in the future. One reason is that bark beetle surveys in the country have by no means been comprehensive, and many areas remain to be explored. For example, *Scolytus carpini* (Ratzeburg, 1837) and *Dryocoetes alni* (Georg, 1856) were both reported as pests on hazelnut in western Russia and may also occur in Turkey (Mandelshtam and Nikitsky 2015, Pomerantzev 1903). *Taphrorychus villifrons* (Dufour, 1843) is common in the Black Sea coastal region and is polyphagous in broad-leaved trees (Mandelshtam and Nikisky 2015). Another reason is that several exotic species have established in the region recently and may spread to Turkey. These include *Xyleborinus attenuatus* (Blandford, 1894), a polyphagous ambrosia beetle now common throughout Europe, and *Scolytoplatypus tycon* Blandford, 1893, introduced to Caucasus (Zamotajlov and Nikitsky 2010). Neither of these species was reported from hazelnuts in Turkey yet, but identifiers and pest managers should be aware of the possibility of their presence.
